# Phenotypic plasticity, QTL mapping and genomic characterization of bud set in black poplar

**DOI:** 10.1186/1471-2229-12-47

**Published:** 2012-04-03

**Authors:** Francesco Fabbrini, Muriel Gaudet, Catherine Bastien, Giusi Zaina, Antoine Harfouche, Isacco Beritognolo, Nicolas Marron, Michele Morgante, Giuseppe Scarascia-Mugnozza, Maurizio Sabatti

**Affiliations:** 1 Department for Innovation in Biological, Agro-food and Forest systems, University of Tuscia, Via S. Camillo de Lellis, Viterbo 01100, Italy; 2 INRA, UR 0588, National Institute for Agricultural Research, Orléans 2 F-45075, France; 3 Department of Agriculture and Environmental Sciences, University of Udine, Via delle Scienze, Udine 33100, Italy; 4 Institute for Mediterranean Agriculture and Forest Systems, National Research Council, Via Madonna Alta, Perugia 06128, Italy; 5 INRA, UMR 1137, INRA-Nancy University, Champenoux F-54280, France; 6 Istituto di Genomica Applicata, Via J. Linussio 51, Udine 33100, Italy; 7 Department of Agronomy, Forestry and Land use, Agricultural Research Council, Via del Caravita, Roma 00186, Italy

## Abstract

**Background:**

The genetic control of important adaptive traits, such as bud set, is still poorly understood in most forest trees species. Poplar is an ideal model tree to study bud set because of its indeterminate shoot growth. Thus, a full-sib family derived from an intraspecific cross of *P. nigra *with 162 clonally replicated progeny was used to assess the phenotypic plasticity and genetic variation of bud set in two sites of contrasting environmental conditions.

**Results:**

Six crucial phenological stages of bud set were scored. Night length appeared to be the most important signal triggering the onset of growth cessation. Nevertheless, the effect of other environmental factors, such as temperature, increased during the process. Moreover, a considerable role of genotype × environment (G × E) interaction was found in all phenological stages with the lowest temperature appearing to influence the sensitivity of the most plastic genotypes.

Descriptors of growth cessation and bud onset explained the largest part of phenotypic variation of the entire process. Quantitative trait loci (QTL) for these traits were detected. For the four selected traits (the onset of growth cessation (date2.5), the transition from shoot to bud (date1.5), the duration of bud formation (subproc1) and bud maturation (subproc2)) eight and sixteen QTL were mapped on the maternal and paternal map, respectively. The identified QTL, each one characterized by small or modest effect, highlighted the complex nature of traits involved in bud set process. Comparison between map location of QTL and *P. trichocarpa *genome sequence allowed the identification of 13 gene models, 67 bud set-related expressional and six functional candidate genes (CGs). These CGs are functionally related to relevant biological processes, environmental sensing, signaling, and cell growth and development. Some strong QTL had no obvious CGs, and hold great promise to identify unknown genes that affect bud set.

**Conclusions:**

This study provides a better understanding of the physiological and genetic dissection of bud set in poplar. The putative QTL identified will be tested for associations in *P. nigra *natural populations. The identified QTL and CGs will also serve as useful targets for poplar breeding.

## Background

Broad-leaved trees grown in temperate zones must avoid periods unfavorable for growth, such as harsh winter weather conditions, synchronizing their annual growth cycle with seasonality. Hence, they have evolved mechanisms to switch between active growth during summer and dormancy during winter, in response to environmental signals.

In trees and woody perennial plants, photoperiod (decrease of day length) typically induces growth cessation, the initiation of cold acclimation, the formation of a terminal bud (bud set) and bud dormancy [1]. Timing and duration of bud set process are inherited as quantitative traits, involving large number of genes with small individual effects [2-4]. Local adaptation of bud set phenology allows trees to find a compromise between the risk of frost damage and the maximization of the growing season duration [5]. In addition, bud activity determines the extent of the seasonal growth, tree architecture, wood quality and productivity [4]. Common garden studies have shown that timing of bud set is strongly correlated with latitude of origin of the population [5-9]. Trees from high latitudes are more sensitive to the photoperiod change than those from southern regions [10]. Even if day length is widely accepted to be the primarily used environmental signal, the timing of bud formation is also influenced by other factors, such as temperature [11], temperature × photoperiod (× population) interaction [12-14], nutrition and drought [4,15]. It has also been demonstrated that bud set in Norway spruce (*Picea abies*) was strongly affected for many years by temperature during zygotic and somatic embryogenesis [16,17], probably influenced by epigenetic memory [18]. This can be seen as a special form of phenotypic plasticity that opens the possibility of adaptation without genetic changes and, therefore, it is an important mechanism when adapting to climate changes [19]. Given the influence of temperature on tree phenology, growth cessation may be affected by climatic warming, which will increase the risk of frost damage in spring, and affect the survival and eventually the spatial distribution of forest trees, because of a less well-suited adaptation to the local altered environment [20]. Since range shift and changes in phenology are the best recorded shifts induced by climate change [19], phenological observations are very important for a better understanding of how different plant species respond to regional climate change [21,22]. Furthermore, research efforts are also required to investigate patterns of phenotypic plasticity, which can represent a crucial determinant of both short- and long-term plant responses [23].

Quantitative trait locus (QTL) mapping is a powerful approach to identify key genomic regions controlling adaptive traits [3,24,25], especially for species where a reference genome is already available [26]. In the face of climate change, the main focus of QTL mapping studies in poplars has been on traits tightly linked to environmental adaptation, such as bud set and bud flush [9,24,27,28]. In *Populus nigra *(black poplar) the genes underlying these adaptive traits remain largely unknown, but genetic maps with hundreds of molecular markers are available [29]. These genetic maps, expanded with gene-specific single nucleotide polymorphisms (SNPs), provide a foundation for the investigation of the genomic regions underlying adaptive traits.

Therefore, the objectives of this study were (i) to assess phenotypic plasticity and evaluate the relative importance of genotype × environment (G × E) interaction in the phenotypic expression (or variation) of bud set in *P. nigra *through a multi-environment approach, (ii) to identify genomic regions underlying the bud set process through QTL mapping and (iii) to select a set of promising candidate genes (CGs) in highlighted genomic regions.

## Results

### Dynamics of bud set process in the two sites

**Figure 1:**
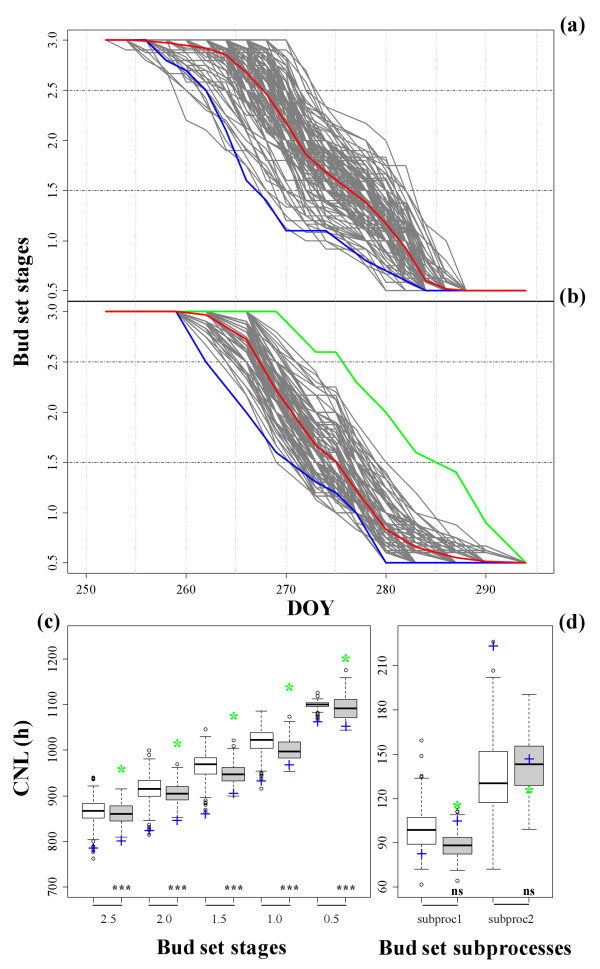
**Phenotypic variation of bud set in a *Populus nigra *full-sib family (POP5)**. POP5 grown in two experimental sites in Italy: Cavallermaggiore (CV) and Viterbo (VT). Genotype means for the progenies in CV (a) and VT (b) are plotted in grey lines. Full-sib family mean is indicated by red lines, the female parent '58-861' by blue lines and the male parent 'Poli' by a green line. Horizontal dashed lines indicate the two critical points of bud set process, which correspond to the onset of growth cessation (date2.5) and the transition from shoot to bud (date1.5). (c) Phenotypic variation for all phenological stages, and (d) subprocesses observed in CV (white boxes) and VT (grey boxes) using block-adjusted genotype means expressed as cumulative night length (CNL). The parent '58-861' is indicated by blue "+", and the parent 'Poli' by green "*". The significance level of differences between the two parents for each trait is indicated as: *ns*, non-significant; *, *P *≤ 0.05; **, *P *≤ 0.01; ***, *P *≤ 0.001.

**Figure 2:**
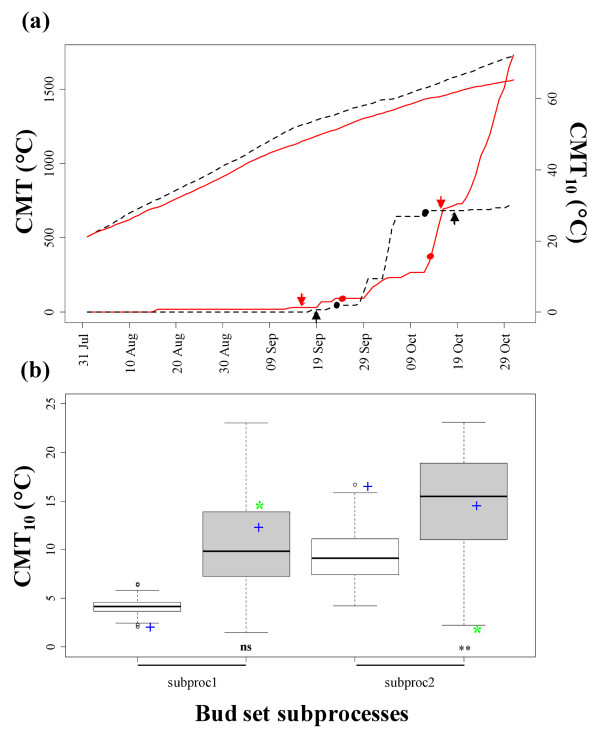
**Temperature and duration of bud set process**. Data were observed in two sites in Italy: Cavallermaggiore (CV) and Viterbo (VT). (a) Cumulative minimum temperature (CMT) and CMT < 10°C (CMT_10_) were calculated from July 1^st ^for CV (red full lines) and VT (black dotted lines). Arrows indicate the two extreme genotypes for bud set stages (date2.5 and date0.5) in CV (red) and VT (black). Dots indicate population mean in CV (red) and VT (black) for the same stages. The upper lines refer to CMT, whereas the bottom lines refer to CMT_10_. (b) Phenotypic variation of subproc1 and subproc2 observed in CV (white boxes) and VT (grey boxes). The parent '58-861' is indicated by blue "+", and the parent 'Poli' by green "*". The significance level of differences between the two parents for each trait is indicated as: *ns*, non-significant; *, *P *≤ 0.05; **, *P *≤ 0.01; ***, *P *≤ 0.001.

The parent '58-861' was the earliest genotype to reach the onset of growth cessation (date2.5) in Viterbo (VT), but it attained this stage beforehand in Cavallermaggiore (CV). In addition, transgressive segregation of some offspring over the earliest '58-861' parent was observed at both field sites (Figure 1a,b,c). However, the parent 'Poli' was the latest genotype to reach all stages of the bud set process. Because 'Poli' is not well-enough adapted to the climate conditions of the CV northern experimental site, it did not survive after re-sprouting. Therefore, significant differences between the two parents for all the onset-of-stage traits could only be detected at VT. Moreover, the two sites did not show clear differences in photoperiod regime during the period of bud set measurements (Additional file 1: Figure S1). Cumulative minimum temperature (CMT) between July and October demonstrated that CV was a colder site on average (Figure 2a). However, when higher weights are given to minimum temperature < 10°C (CMT_10_), the observed cumulative minimum temperature pattern provided additional description of a temporary but early fall of temperature at VT.

The average timing of date2.5 at population level was similar at the two sites, i.e. day of the year (DOY) 267 amounting to about 864 and 862 h of cumulative night length (CNL) in CV and VT, respectively, with only three minutes difference in terms of effective night length between the two sites (Table 1). The same population mean was also found at both sites for date0.5, i.e. DOY 286 (corresponding to a minor difference in terms of CNL), for a total of 18.8 d needed on average by the full-sib family studied (POP5) to cover the whole phenological process (Table 1). Actually, the high value of Pearson correlations among the five phenological stages showed that, when the growth cessation process begins, all the onset-of-stage traits follow constantly through a cascade process (Table 2a,b). While the population mean of the most distant phenological stages was the same at both sites, a great variation at genotypic level could be observed. Early bud set genotypes reached date2.5 at DOY 262.9 in VT and at DOY 259 in CV, with about four days differences between sites.

**Table 1:** Timing and duration of bud set process in a *Populus nigra *full-sib family (POP5).

Population mean										Genotype mean					
Site	date2.5			date0.5			Total duration of process			Earliest for date2.5			Latest for date0.5		
	
	DOY	CNL (h)	CMT_10 _(°C)	DOY	CNL	CMT_10_	DOY	CNL	CMT_10_	DOY	CNL	CMT_10_	DOY	CNL	CMT_10_
**CV**	267.65	864.23	3.70	286.45	1098.25	17.29	18.80	234.02	13.59	259.03	762.21	1.49	288.54	1125.65	22.57
**VT**	267.32	861.95	2.76	286.17	1093.54	27.82	18.85	231.59	25.06	262.87	809.51	0.46	292.29	1175.05	28.51

POP5 grown in two experimental sites in Italy: Cavallermaggiore (CV) and Viterbo (VT). Values are expressed as day of the year (DOY), cumulative night length (CNL) and cumulative minimum temperature < 10°C (CMT_10_) for date2.5, date0.5 and the total process length at population level. The genotype means of date2.5 for the earliest genotype and of date0.5 for the latest one, expressed using all the parameters considered, are shown.

**Table 2:** Correlations between bud set traits in a *Populus nigra *full-sib family (POP5).

	(a)							(b)						
	**date2.5**	**date2**	**date1.5**	**date1**	**date0.5**	**subproc1**	**subproc2**	**date2.5**	**date2**	**date1.5**	**date1**	**date0.5**	**subproc1**	**subproc2**

date2.5		0.97	0.89	0.81	0.62		-0.84		0.98	0.93	0.83	0.63		-0.24

		***	***	***	***		***		***	***	***	***		**

date2	0.99		0.96	0.89	0.61		-0.92	0.99		0.98	0.90	0.69		-0.20

	*0.00*		***	***	***		***	*0.00*		***	***	***		*

date1.5	0.93	0.97		0.97	0.63	0.24	-0.96	0.98	1.00		0.96	0.76	0.36	-0.12

	*0.01*	*0.00*		***	***	**	***	*0.00*	*0.00*		***	***	***	*ns*

date1	0.87	0.93	0.98		0.68	0.33		0.96	0.99	1.00		0.88	0.50	

	*0.02*	*0.01*	*0.00*		***	***		*0.01*	*0.00*	*0.00*		***	***	

date0.5	0.78	0.72	0.58	0.84		0.03		0.88	0.93	0.96	0.98		0.49	

	*0.03*	*0.04*	*0.05*	*0.02*		*ns*		*0.02*	*0.01*	*0.01*	*0.00*		***	

subproc1			-0.08	-0.17	0.11		-0.27			-0.31	-0.42	-0.59		0.28

			*0.08*	*0.08*	*0.08*		***			*0.07*	*0.07*	*0.05*		***

subproc2	-0.92	-0.98	-1.00			0.08		-0.56	-0.47	-0.37			0.74	

	*0.01*	*0.00*	*0.01*			*0.08*		*0.06*	*0.06*	*0.07*			*0.04*	

POP5 grown in two experimental sites in Italy: Cavallermaggiore (CV) and Viterbo (VT). Data were analyzed on cumulative night length (CNL) basis. (a) Within site phenotypic (above the diagonal) and genetic (below the diagonal, with ± standard deviation (SD)) correlations for CV and (b) likewise for VT. The levels of significance of the Pearson coefficient are indicated as: *ns*, non-significant; *, *P *≤ 0.05; **, *P *≤ 0.01; ***, *P *≤ 0.001.

As for bud set subprocesses at the two sites, the duration of bud maturation (subproc2, 10.6 d in CV and 11.6 d in VT) was longer than the duration of bud formation (subproc1, 8.2 d in CV and 7.3 d in VT). Consequently, subproc2 experienced longer CNL and higher CMT_10 _than subproc1 (Figure 1d and 2b). Moreover, subproc2 was negatively correlated with date2.5 especially at the northern site (r = -0.84) (Table 2a).

In VT, phenological patterns of the two parents did not show significant differences in subprocesses relating to CNL (Figure 1d). With regards to CMT_10_, differences were exhibited only in subproc2 (Figure 2b). The parent '58-861' was one of the fastest genotypes to complete subproc1 in CV but, on the contrary, it was very slow to complete subproc2. However, in VT, the two parents did not show extreme values in the family distribution and scored similar subprocesses in terms of CNL (Figure 1d). For the parent 'Poli', subproc1 and subproc2 showed similar duration relating to CNL in VT (Figure 1d), but it experienced cold temperature only in subproc1 (Figure 2b). The parent '58-861' required more time to complete subproc1 and less time to complete subproc2 in VT than in CV (Figure 1d). Differences in subproc1 dependent on CMT_10 _were observed between the two sites (Figure 2b). Linear regressions between selected phenological characters, according to principal component analysis (PCA), and biometric traits demonstrated that in our experiments growth characters did not influence the phenotypic variation of bud set (Additional file 2: Figure S2).

### Broad-sense heritability of bud set traits

Substantial within-site genetic variation for all traits is presented in Additional file 3: Table S1 and Additional file 4: Table S2. In general, data showed higher levels of phenotypic variance and lower broad-sense heritability (H^2^) for duration traits as compared to onset-of-stage traits (Table 3). Phenological bud set traits expressed as CNL showed a decreasing trend in terms of genetic component of variance from date2.5 to date0.5 at both sites, with broad-sense heritability at genotypic level (H_gen_^2^) ranging from 0.84 to 0.43 in CV, and from 0.74 to 0.45 in VT (Table 3). The transition from shoot to bud structure (i.e. date1.5) showed quite similar H_gen_^2 ^values between the two sites (0.73 < H_gen_^2 ^< 0.75). Duration traits and subprocesses, analysed using the CNL parameter, showed low levels of H^2 ^especially at individual level (H_ind_^2^) in VT (0.03 < H_ind_^2 ^< 0.09) (Additional file 4: Table S2).

**Table 3:** Variance components and genotype ranking for stages and duration traits of bud set process.

CNL	Variance components (%)							Broad-sense heritability		Spearman Rank	
			
	σ^2^_E_/σ^2^_P_		σ^2^_G_/σ^2^_P_		σ^2^_G×E_/σ^2^_P_		σ^2^_ε_/σ^2^_P_	H^2^_gen_ CV	H^2^_gen_ VT	ρ	
date2.5	0.0	*ns*	29.6	***	19.0	***	51.4	0.84	0.73	0.51	***
date2	1.1	***	27.1	***	18.8	***	53.0	0.81	0.74	0.51	***
date1.5	5.6	***	21.8	***	16.8	***	55.8	0.75	0.73	0.46	***
date1	9.7	***	17.2	***	13.1	***	60.0	0.70	0.64	0.39	***
date0.5	2.9	***	5.8	***	9.2	***	82.1	0.43	0.45	0.22	**

duration2.5	3.2	***	8.9	***	0.0	*ns*	87.9	0.36	0.28	0.27	**
duration2	14.5	***	2.9	***	7.7	**	74.8	0.49	0.24	0.09	*ns*
duration1.5	6.3	***	0.9	***	11.5	***	81.3	0.57	0.15	0.06	*ns*
duration1	12.4	***	1.9	***	16.3	***	69.4	0.62	0.10	0.08	*ns*

subproc1	11.3	***	5.3	***	4.9	*	78.4	0.44	0.27	0.18	*
subproc2	3.1	***	0.8	***	20.0	***	76.1	0.67	0.15	0.01	*ns*

Data were obtained from a *Populus nigra* full-sib family (POP5) grown in two sites in Italy: Cavallermaggiore (CV) and Viterbo (VT). Data were analyzed on cumulative night length (CNL) basis. Relative importance of environment (σ^2^_E_), genotype (σ^2^_G_), genotype by environment (σ^2^_G×E_) and residual (σ^2^_ε_) effects on the phenotypic variation (σ^2^_P_) are presented together with values of broad-sense heritability at genotypic level (H^2^_gen_). Spearman rank coefficients (ρ) were calculated from genotype means in CV and VT. The significance level of differences between the two parents for each trait is indicated as: ns, non-significant; *, *P *≤ 0.05; **, *P *≤ 0.01; ***, *P *≤ 0.001.

We observed a positive genetic correlation among onset-of-stage traits. Additionally, subproc2 was negatively correlated with date2.5 (r_g _= -0.92 ± 0.01 in CV and r_g _= -0.56 ± 0.06 in VT). Both these cases were also previously shown by linear correlations at the two sites (Table 2a,b).

### G × E interaction and phenotypic plasticity

Genotype × environment (G × E) interaction was calculated using a two-way analysis of variance with environment (including both site and year) and genotype as variation factors. The sources of phenotypic variation (genotype, G × E) were statistically significant with no environment effect in date2.5. Likewise, there was no G × E interaction effect on duration2.5 (Table 3). The relative importance of the genotypic effect as well as the G × E component were considerable in all the onset-of-stage traits with a decreasing trend from date2.5 to date0.5. The phenotypic variation explained by the genotype component ranged from 29.6% (date2.5) to 5.8% (date0.5) and from 19.0% (date2.5) to 9.2% (date0.5) for the G × E interaction.

Spearman rank coefficients were positive, moderate and highly significant for the onset-of-stage traits, but they decreased during time. Hence, there were not significant changes in the ranking of genotypes between environments, but the degree of changes increased gradually during time (Table 3). Concerning duration traits, low levels of genotypic variance (0.8% < σ_G_^2^/σ_P_^2 ^< 8.9%) and high levels of residuals (up to 87.9%) in the phenotypic variation were observed, with an increasing importance of the G × E interaction during bud development (from 0% to 20%). The low values of Spearman coefficients for duration traits were a clear indication of strong and random changes in genotype ranking between environments (Table 3).

**Figure 3:**
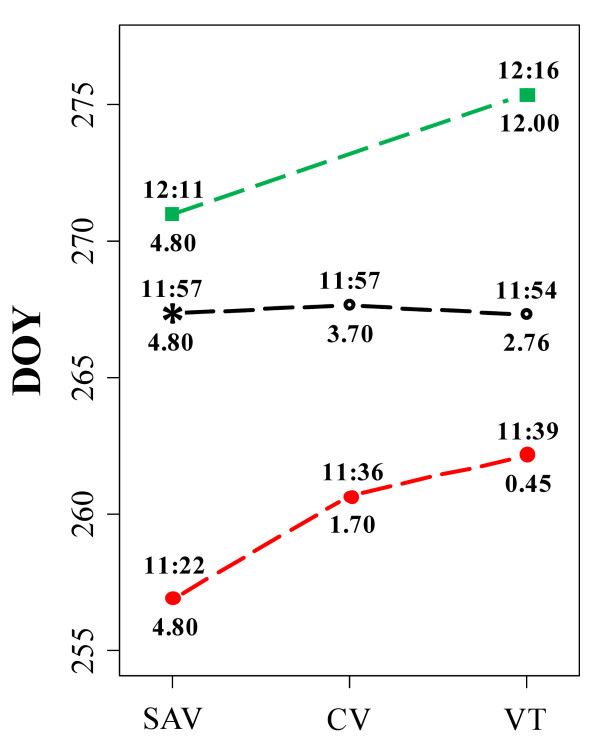
**Interaction pattern (i.e. the genotype-specific environment-phenotype function) for the onset of growth cessation (date2.5)**. Data were recorded as day of the year (DOY) in a full-sib *Populus nigra *family (POP5) grown in different sites in Italy: Cavallermaggiore (CV), Viterbo (VT) and Savigliano (SAV). The red dots indicate the female parent '58-861' that is native to North of Italy. The green squares indicate the male parent 'Poli' that is native to South of Italy. The black open dots indicate the mean of the full-sib family. A set of seven genotypes from POP5 was grown and scored in SAV and the corresponding value is indicated by "*". Numbers above and below each line represent the effective night length (h) and the cumulative minimum temperature < 10°C (CMT_10_), respectively.

The interaction pattern of the population mean for date2.5 expressed as DOY, indicative of the critical day length inducing growth cessation, showed an almost horizontal trend for the three studied sites. Parent '58-861' was a very plastic genotype showing an earlier onset of growth cessation in Savigliano (SAV) and CV as compared to VT. The onset of growth cessation was initiated at different night lengths at the three sites, with a difference of about 20 min between SAV and VT. CMT_10 _in SAV, CV and VT (4.8°C, 1.7°C and 0.45°C, respectively) contributed to the delay of growth cessation of this genotype (Figure 3). Conversely, 'Poli' appeared to be less plastic for date2.5 and its response seemed mainly influenced by the photoperiod. It reached date2.5 at a similar effective night length, with a difference of 5 min between SAV and VT, showing a minor sensitivity to increasing value of CMT_10 _(Figure 3).

**Figure 4:**
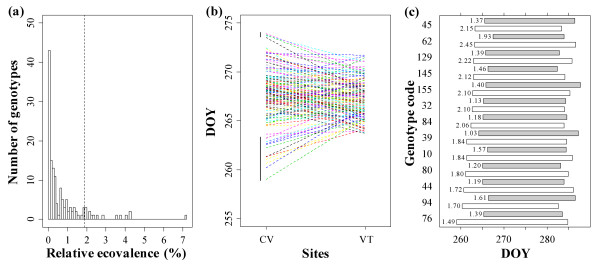
**Analysis of phenotypic plasticity of bud set in a *Populus nigra *full-sib family (POP5)**. Data were observed in two sites in Italy: Cavallermaggiore (CV) and Viterbo (VT). (a) Frequency distribution of the genotype relative ecovalence (%). The vertical dotted line separates the 17 most plastic genotypes, accounting 50% of the total G × E observed for the onset of growth cessation (date2.5). (b) Interaction patterns of the full-sib genotypes. The vertical black lines indicate the 17 most plastic genotypes. (c) Total duration of bud set process for 13 of these plastic genotypes that showed an interaction pattern with a positive slope CV-VT in terms of cumulative minimum temperature < 10°C (CMT_10_). Bars represent the duration from onset of growth cessation (date2.5) to the end of bud maturation (date0.5), measured in day of the year (DOY) in CV (white bars) and VT (grey bars). The CMT_10 _value corresponding to date2.5 is reported on the left of each bar.

The relative ecovalence analysis (Figure 4a) showed only a few genotypes contributing to the overall G × E interaction. These genotypes were the most extreme genotypes in CV for date 2.5 (Figure 4b,c). Seventeen of the most plastic genotypes contributed for 50% of the G × E interaction. Thirteen of them demonstrated an interaction pattern describing a positive slope CV-VT in relation to CMT_10_. The remaining four genotypes, which were the latest in CV, had a negative slope that was probably due to non controlled factors (e.g. site quality and nutrients availability) (Figure 4b). The duration of the bud set process of the thirteen plastic genotypes with early date2.5 in CV was always shorter in VT than in CV. Moreover, these genotypes always initiated date2.5 later in VT than in CV, where CMT_10 _was reached earlier (Figure 2a and Figure 4c).

### QTL analyses

**Figure 5:**
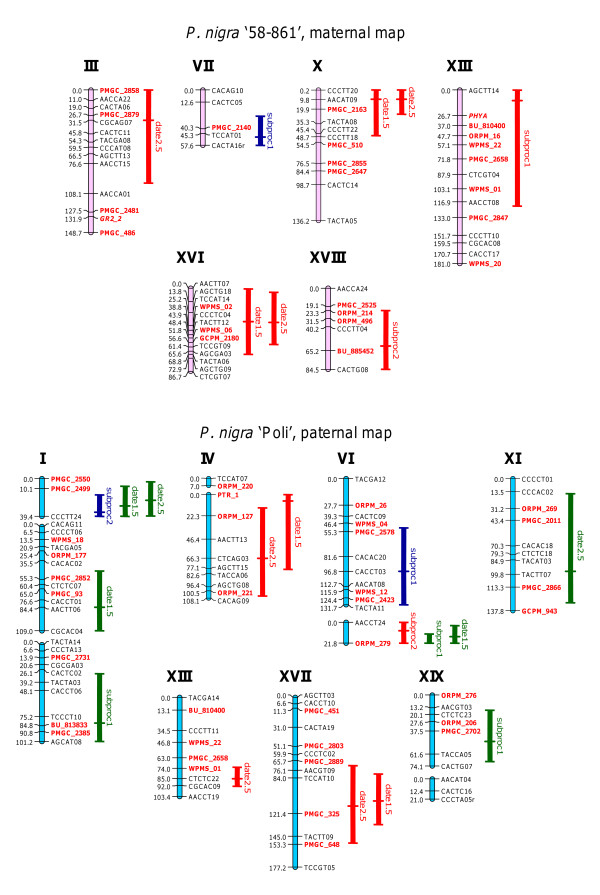
**Quantitative trait loci (QTL) for bud set**. Data were obtained from a *Populus nigra *full-sib family (POP5) grown in two sites in Italy: Cavallermaggiore (CV) and Viterbo (VT). Selected traits related to bud set phenology (onset-of-stage traits: date2.5 and date1.5; duration traits: subproc1 and subproc2) were considered. Roman numerals over the linkage groups (LGs) correspond to the *P. trichocarpa *chromosomes. LGs of maternal map (genotype '58-861') are in pink and LGs of paternal map (genotype 'Poli') are in blue. The length of the LG bars is proportional to the map distance in centiMorgan (cM). Numbers on the left of each LG indicate the marker position in cM. Amplified Fragment Length Polymorphism (AFLP) markers are in black and Simple Sequence Repeats (SSRs) are in red and in bold. QTL are represented on the right side of the LGs by lines that indicate the 95% confidence interval. LOD peaks are marked by a short horizontal tick. Red lines denote QTL with a positive genetic effect in CV and VT. Green lines denote QTL with a negative genetic effect in CV and VT. Blue lines denote QTL with an opposite effect between CV and VT.

Discriminative bud set traits for QTL analysis were a list of complementary traits, which explain together a large part (% from PCA on this list of traits) of phenotypic variation observed for growth cessation and bud onset. The trait date2.5, although highly correlated with date1.5, was added because we consider it physiologically remarkable (perception of critical day length) and scarcely reported in literature. For these four traits eight and 16 QTL were mapped on the maternal and paternal map, respectively. Genetic parameters for the detected QTL are shown in Table 4. The QTL intervals were located on 12 linkage groups (LG): I, III, IV, VI, VII, X, XI, XIII, XVI, XVII, XVIII and XIX (Figure 5). QTL for date1.5 and date2.5 co-localized on LG-IV, X, XVI, XVII, and on LG-Ia together with subproc2. On LG-VIb, QTL intervals for subproc1, subproc2 and date1.5 overlapped (Figure 5). The average percentage of variance explained (PVE) by individual QTL was 5.5%, and the maximum PVE per trait was 40.1% (Table 5). Three QTL, two for subproc1 and one for subproc2, had effects with opposite directions between the two sites (Table 4), thus reflecting the negative genetic correlation between these traits and the onset-of-stage traits (Table 2a,b). Seven of the 16 QTL for the paternal map and one of the eight QTL for the maternal map had significant differences between the two sites (Table 4).

**Table 4:** Identified quantitative trait loci (QTL) for bud set.

Parental map	Trait	LG	LOD	95% CI	Genetic effect		*P-*value	PVE	
							
					CV	VT		CV	VT
Poli	date2.5	Ia	3.46	[3.2-39.1]	-21.576	-2.912	***	0.119	0.004

Poli	date1.5	Ia	3.31	[7.9-39.4]	-19.869	-3.091	**	0.099	0.004

Poli	subproc2	Ia	3.24	[16.98-39.4]	19.448	-0.894	*	0.126	0.001

Poli	date1.5	Ib	3.14	[46.4-109]	-12.802	-8.896		0.041	0.036

Poli	subproc1	Ic	3.58	[30.4-101.1]	-9.750	-1.187	*	0.099	0.005

Poli	date2.5	IVb	4.15	[13.8-105.9]	13.455	11.056		0.046	0.064

Poli	date1.5	IVb	4.64	[0-78]	19.031	11.489		0.091	0.059

Poli	subproc1	VIa	2.50	[51.4-131.6]	-2.462	4.706	*	0.006	0.072

Poli	date1.5	VIb	4.50	[3.4-21.8]	-22.109	-1.114	***	0.126	0.001

Poli	subproc1	VIb	2.91	[12.1-21.8]	-8.473	-1.438		0.075	0.007

Poli	subproc2	VIb	2.59	[0-21.8]	17.730	1.219	*	0.104	0.001

Poli	date2.5	XI	2.94	[15.7-129.5]	-13.841	-9.081		0.049	0.043

Poli	date2.5	XIII	4.81	[72.8-92.9]	16.009	9.950		0.065	0.051

Poli	date2.5	XVII	3.30	[71.3-152.1]	10.145	12.910		0.026	0.085

Poli	date1.5	XVII	4.11	[79.8-132.6]	13.047	15.258		0.044	0.105

Poli	subproc1	XIXa	2.32	[15.8-69.4]	-5.019	-4.323		0.026	0.061

58-861	date2.5	III	3.40	[0-97.1]	3.761	13.106		0.003	0.085

58-861	subproc1	VII	2.77	[27.3-57.7]	-0.107	5.385		0.000	0.093

58-861	date2.5	X	5.80	[0-25]	16.742	14.312		0.065	0.102

58-861	date1.5	X	4.95	[0-47.4]	12.925	16.204		0.038	0.113

58-861	subproc1	XIII	2.49	[0.2-121.1]	10.093	0.839	*	0.107	0.002

58-861	date2.5	XVI	3.85	[4.2-58.6]	18.767	8.545		0.081	0.036

58-861	date1.5	XVI	2.84	[0.7-68.9]	17.008	8.341		0.066	0.030

58-861	subproc2	XVIII	2.20	[23.2-84.5]	9.369	8.932		0.028	0.057

Data were obtained from a *Populus nigra *full-sib family (POP5) grown in two sites in Italy: Cavallermaggiore (CV) and Viterbo (VT). Selected traits related to bud set phenology (onset-of-stage traits: date2.5 and date1.5; duration traits: subproc1 and subproc2) were considered. For each QTL, the parental map, the linkage groups (LG), the logarithm of the odds (LOD), the 95% confidence interval (CI) in cM, the genetic effect and the percentage of phenotypic variance explained (PVE) at CV and VT, are shown. *P*-values indicate significant site effect: *, *P *≤ 0.05; **, *P *≤ 0.01; ***, *P *≤ 0.001. PVE values ≥ 0.1 are underlined.

**Table 5:** Phenotypic variance explained by quantitative trait loci (QTL).

Trait	Number of QTL		Total PVE (%)			
			
			58-861		Poli	
	
	58-861	Poli	CV	VT	CV	VT
date2.5	3	5	**14.9**	**22.3**	30.5	**24.7**
date1.5	2	5	10.4	14.3	**40.1**	20.5
subproc1	2	4	10.7	9.5	20.6	14.5
subproc2	1	2	2.8	5.7	23.0	0.2

Total percentage of phenotypic variance (%) explained (PVE) by the QTL per trait and per site for the two *Populus nigra *parents 'Poli' and '58-861'.

Maximum values per genotype and site are in bold.

### Identification of CGs

Anchored simple sequence repeat (SSR) markers have allowed linking 19 out of 24 identified QTL to the physical map. Genomic intervals of QTL on LG-I, IV, X, XIII, XVI and XVII, corresponding to co-locating QTL with high PVE, were analyzed. Their intervals ranged from 1.7 to 10.6 Mbp, considering a unique overlapping region where QTL co-localized. The number of gene models in these intervals ranged from 125 to 990. The total number of gene models in all the intervals was 3317 (Table 6). The availability of the genome sequence of the parental genotype 'Poli' and three other *P. nigra *genotypes [30], allowed the calculation of the number of SNPs in each interval for 'Poli' and among the four *P. nigra *genotypes (Table 6).

**Table 6:** Selected quantitative trait loci (QTL) regions associated with bud set in *Populus nigra*.

Parent - LG	Traits	Flanking SSRs	Overlap QTL interval length		Position on *P. trichocarpa *v2 (kbp)		Genes and SNPs within QTL interval			**722 CGs from Ruttink *et al ***[31]		CGs from other sources	
			**cM**	**Kbp**	**Start**	**End**	**Genes**	**Poli SNPs**	intrasp. SNPs*	**all LG**	**within QTL**	**all LG**	**within QTL**

Poli - Ia	date2.5	PMGC_2550-PMGC_2499	22.12	1663.095	4828.200	6491.295	217	2967	14482	81	2	4	0
													
	date1.5												
													
	subproc2												

Poli - IV	date2.5	PTR01-ORPM_221	64.2	7780.103	8083.898	15864.000	470	29159	88907	39	6	4	2
													
	date1.5												
													
	date1.5												

Poli - XIII	date2.5	PMGC_2658-WPMS_1	20.1	5524.000	6787.986	12312.018	322	17536	57215	42	5	1	0

Poli - XVII	date2.5	PMGC_2889-PMGC_325	52.8	6159.403	8295.993	14455.396	445	23391	99316	21	7	2	1
													
	date1.5												

58-861 - X	date2.5	PMGC_2163-PMGC_510	25	2839.533	3827.925	6667.458	125	11418	33742	46	2	4	0
													
	date1.5												

58-861 - XIII	subproc1	WPMS_02-GCPM_2180	120.9	10565.891	0.001	10565.891	954	30973	100689	42	29	1	1

58-861 - XVI	date2.5	WPMS_02-GCPM_2180	34.89	8956.860	0.001	8956.861	990	27957	94980	31	20	2	2
													
	date1.5												

QTL intervals were projected on the physical genome sequence by anchored markers (flanking SSRs). The length of the QTL intervals in centiMorgan (cM) and in Kilobase pairs (Kbp) is given together with the number of gene models, single nucleotide polymorphisms (SNPs) for the male parent 'Poli' and SNPs between 'Poli' and three other *P. nigra *genotypes (71077-308, BEN3, and BDG) [30]. The table also reports the number of candidate genes (CGs) for bud development from Ruttink et al. [31] and other sources (literature and GenBank) on linkage groups (LG) containing QTL and within the QTL interval.

* SNPs among four *P. nigra *genotypes (Poli, 71077-308, BEN3, and BDG).

QTL analyses cannot identify specific genes responsible for the phenotypic variation, but the integration of genomic data with the knowledge of gene function or expression allowed identifying promising CGs linked to bud set. Literature searches about phenology, coupled with the search of GenBank database, allowed finding a total of 997 genes reported to be involved in bud set. Among them, 774 sequences were located on the *P. trichocarpa *genome assembly v2 (corresponding to 1.9% of all *P. trichocarpa *genes), 722 were identified by Ruttink et al. [31], and 52 from other sources (Additional file 5: Table S3). Sixty seven expressional CGs from Ruttink et al. [31] and six from the other sources were within the seven QTL regions analyzed, corresponding to 2.2% of all genes contained in these intervals (Table 6 and Additional file 5: Table S3). The latter set of six CGs were: one photoreception factor gene, *Phytochrome A *(*PhyA*, GI: 2664188); three transcription factor genes for signal transduction, *Constans2-like *(*Co-L2*, GI: 831441), *FAR-RED elongated hypocotyl 1 protein *(*FHY1.2*), and *PhyA signal transduction factor *(*PAT1-LGXVI*); two circadian clock factor genes, *circadian clock coupling factor *(*ZGT1like*, GI:14210078 and *ZGT2like*, GI:14210078). Since these six candidates did not come from expressional evidence, a search in the available expressed sequence tag (EST) database (*Populus*DB, GenBank and others) was conducted in order to find evidence of expression. All the six gene sequences showed significant hits vs. ESTs belonging to a mixed tissue preparation of *Populus *leaf, bud and stem, which could be referred to as phenology-related tissues. In the *P. nigra *mapping population, a single marker *t*-test (Kruskal-Wallis) between phenotypic data of subproc1 and SNP polymorphism used to map *PhyA *indicated that this SNP was not responsible for the effect of subproc1 QTL (data not shown). Furthermore, gene models were investigated closest to the logarithm of the odds (LOD) peak of selected QTL, considering ± 10 kbp from the LOD peak position [32]. When no gene was found, the region was extended to ± 30 kbp to find at least one gene per LOD peak. Eighteen gene models corresponding to these search criteria were found and listed in Additional file 6: Table S4. Some of these genes were functionally involved in regulation of transcription (putative transcription factors *MYB6*, *BNQ1*, *bHLH*), intracellular signal transduction (*DC1 domain-containing protein*), photomorphogenesis (*CULLIN4*) and UV light sensitivity (*UVI4*). The latter gene is also present in the list of expressional CGs found by Ruttink et al. [31]. Other genes close to LOD peaks were related to developmental process (*CRINKLY4 RELATED*, *RABA4D*), response to stress and/or hormones (*AMK2*, *CCR4-NOT*, *ACR8*) and others encoded structural constituent of ribosome (Additional file 6: Table S4). The EST database search revealed that 16 out of 18 gene models are putatively expressed. Among them, 13 models showed significant hits matching to EST libraries belonging to tissues related to phenology (i.e. bud, flower, apical shoot).

Given the data collected in terms of QTL co-localization, expression and literature reports, we came up with a set of most promising CGs for bud set traits composed of six functional candidates, 13 gene models and 67 expressional candidates.

## Discussion

### Unravelling bud set process and G × E interaction

*Populus *is an ideal model tree to study bud set because of its indeterminate shoot growth. Another factor worthy of consideration is that POP5 parental genotypes originated from contrasting environments. These two advantages render powerful the exploration of the G × E interaction patterns and phenotypic plasticity that are mostly uncharacterized for phenology traits. Recently, a new bud set scoring system in *Populus *spp. has led to the dissection of the bud set process into different stages and durations to estimate their relative contribution to the accomplishment of this phenological process [9]. Based on this new tool, the phenotypic plasticity and the G × E interaction was fully investigated in a *P. nigra *F_1 _full-sib family using a multi-environment approach. The transition from shoot to bud structure (date1.5) was selected as the very critical determinant of bud set at both sites by PCA analysis. The selection of phenotypic descriptors characterizing key steps of bud set process allows to improve the estimation of genetic variation expressed at these different steps and the detection of more robust genomic regions involved in the phenological process. High phenotypic and genetic correlations between stage traits, consistent to those observed in *P. nigra *natural population [9], pointed out bud set as a cascade process, during which one phase could be indicative of the whole process. Genetic correlations between the two subprocesses vary in magnitude and sign from one environment to another. Indeed, in the past it was recognized that genetic correlations change across environments [33].

In the present study, the timing of stages and duration traits showed different levels of H^2^. This contrast could be explained simultaneously by different levels of expressed genetic variation and different levels of residual variation. Stage traits were characterized by a significant genotypic effect and a high heritability value, but the influence of the residual factors, either environmental conditions or precision of measure, augmented from date2.5 to date0.5. The increase of the importance of environmental factor from date2.5 to date0.5 is contrary to the one observed by Rohde et al. [9] in hybrid poplars, but similar to the one observed in *P. nigra *natural populations studied in the same paper. A major genetic control of the last part of the process (date1, the only scored stage) was also found in a F_2 _hybrid poplar pedigree by Howe et al. [34]. Similar results were also found by Li et al. [35] in interspecific poplar hybrids in two successive years. This major H^2 ^may be explained by the use of interspecific hybrids that could increase the genetic variance and heritability estimation for these traits [34]. Nevertheless, heritability estimates have often been shown to be specific to the population and environment [36,37].

The total duration of the bud set process for POP5 was conserved at the two sites, but the time spent in each subprocess depended on the site. However, in both cases subproc2 was longer than subproc1. Interestingly, as exhibited by phenotypic correlations we observed that the later genotypes for date2.5 hastened subproc2, which could be an important strategy to avoid frost injury.

According to the concept of critical night length, where photoperiod is responsible for the onset of bud set process across environments and years [38], POP5 family reached date2.5 at about the same effective night length at both sites. Importantly, ANOVA analysis for date2.5 showed a non-significant contribution of the environment effect and, on the opposite, a significant G × E effect (up to 19% of the phenotypic variability explained). This indicates that genotypes react differently to different environments. Data showed always a highly significant contribution of the G × E interaction in the phenotypic variation of all the stages, with a decreasing trend observed during the process. Rohde et al. [14] demonstrated that different locations differentially influenced the timing of growth cessation and the duration of bud formation in identical poplar genotypes, suggesting that high temperatures render the meristem less sensitive to growth-arresting photoperiodic signals. Moreover, Kalcsits et al. [11] showed that night temperature has a greater influence than day temperature in poplar. In the present study, the growth cessation of the plastic genotypes started earlier at the site with the lower minimum overnight temperature. Total duration of the bud set process for plastic genotypes was hastened in VT where lower temperatures were experienced during bud set. The site VT was globally warmer than CV with higher temperatures prevailing during the time until growth cessation, but the fall of temperatures recorded in VT during a limited number of nights was like a short treatment in an usually warmer site. Few recent studies showed that temperature can modify the timing of photoperiod controlling phenological events in deciduous woody plants [11,13,39]. The relative contribution of the G × E interaction on the phenotypic variation observed on date1 was perfectly comparable with the results of Luquez et al. [5] in *P. tremula *in two sites. In our *P. nigra *family the magnitude of G × E interaction partially increased from subproc1 to subproc2. The latter is a developmental period for which the genotypes of the analyzed family tended to respond differently to the various environments.

Distribution of genotype relative ecovalences showed that only few genotypes strongly contributed to the overall G × E interaction for date2.5. More than important rank changes, these genotypes revealed differences in the scale of response to environmental changes. This is the case for '58-861', one of the earliest genotype for the onset of growth cessation (date2.5) at both sites but sensitive to cold temperatures. This could be an important survival strategy for trees to adjust their short-term response to temperature in a changing climate. Increasing rank change effect responsible for the G × E interaction was observed during the bud set process. Non-significant Spearman coefficients indicated random changes in genotype ranking for duration traits. Consequently, a major influence of environmental conditions on the variation of these traits was observed.

### Genomic regions containing QTL for bud set

The QTL detected in this study explain a small proportion of the phenotypic variance (≤ 10%), which is likely overestimated due to the relatively small progeny size [15]. However, several studies on global gene expression demonstrated the complex gene networks involved in bud formation, with large sets of genes temporally regulated during each phase of the process [31,40,41]. Therefore, one can expect a complex genetic control of bud formation by many genes with minor effect on the phenotype. Similar results were also described by Jermstad et al. [42] for spring bud flush in Douglas-fir. In forest trees, biological and technical constraints make difficult to manage wide progeny sizes, thus limiting the complete genome coverage with high resolution genetic maps. This negatively affects the detection of QTL with major effect, if present within uncovered genome regions, and reduces possibility to detect QTL with minor effect. The number of QTL identified also depends on the importance of genetic factors for the trait. This is in agreement with our observations, with more QTL identified for the onset-of-stage traits that showed medium to high H^2 ^than for the duration traits.

The co-localization of most QTL for stages and subproc2 was in agreement with their high genetic correlation, relating to the physiological relationship between these traits. This could be due to the pleiotropic effect of a single gene affecting the traits or the physical linkage and/or linkage disequilibrium between two loci influencing the traits [43]. Negative genetic correlations indicate antagonistic effects of the loci on the traits. When stages QTL co-localized with the subproc2 QTL, they had opposite genetic effect.

Seven QTL on 'Poli' map and one on '58-861' map have significantly different effects between the two environments, underlying the significant G × E interaction. Phenotypic data indicated that genotype '58-861' was highly plastic, while 'Poli' encountered difficulties in adapting to the CV site. This suggests that the expression of alleles inherited from the 'Poli' parent is more dependent on the environment than the expression of alleles inherited from '58-861' parent.

Regardless of the slight changes in the genetic maps and the addition of a second site (VT), the QTL with high PVE were identified as in Rohde et al. [9], who analyzed the same mapping family but with less individuals. When the framework map was compared to that described by Gaudet et al. [29] and then used by Rohde et al. [9], the addition of new F_1 _individuals has led to the mapping of new framework markers and to the splitting of some LGs. However, QTL were detected on LG-III, LG-VI and LG-XIII, which belong to the six robust regions selected by Rohde et al. [9]. Previously, QTL for bud set were identified in another pedigree of *P. trichocarpa *× *P. deltoides *on LG-III, VI and X [24]. These three LGs have also been detected in Rohde et al. [9] and in the current study. The genes *PhyB2*, *LHY1 *and *LHY2*, of which SNP polymorphism explain part of the phenotypic variation of bud set in *P. tremula *[44,45], were not within the common QTL intervals across several *Populus *mapping pedigrees [9,24]. QTL regions recurrently identified in different pedigrees and environments are most likely robust genomic regions controlling the variation of the traits.

### Identifying positional candidate genes

QTL mapping is an early step in identifying genes underlying trait variation [43]. The availability of the *P. trichocarpa *genome sequence adds a new dimension to QTL mapping, allowing to investigate genes within QTL intervals and map CGs *in silico*. Deep analysis of the most robust QTL regions allowed to highlight 13 gene models, 67 bud set-related expressional CGs and six functional CGs (*PhyA*, *Co-L2*, *FHY1.2*, *PAT1*, *ZGT1like *and *ZGT2like*).

The functional CGs are involved in light signaling pathway. In particular, *Co-L2*, that belongs to the *CONSTANS *gene family, was found to be regulated by photoperiod and involved in growth cessation in Norway spruce [46] and *Populus *[47,48]. *PAT1*, *FHY1.2*, *ZGT1like *and *ZGT2like *are also involved in the regulation of *PhyA *signaling [49-51], which could affect the quantitative variation of bud set. This regulation mechanism could explain why *PhyA *was not directly responsible for the phenotypic variation of this trait (Kruskal-Wallis single marker *t*-test), even if it is well known to play a key role in growth cessation [52].

Another interesting CG is the *UV-B light insensitive 4 *(*UVI4*), which is close to a peak of QTL LOD and also belongs to the expressional CGs found by Ruttink et al. [31]. *UVI4 *is responsive to UV-B light and its expression level decreased with the cessation of cell division in *Arabidopsis thaliana *[53]. In *Populus*, *UVI4 *gene was also found to be down-regulated during bud formation and cambial dormancy [31].

Among the hundred gene models annotated within our large QTL intervals, we investigated those close to peaks of LOD. The identified CGs that are then supported by *in silico *evidences of expression in phenology-related tissues, mainly respond to light and cold as the functional CGs within QTL intervals. Therefore, they could participate to environmental adaptation by the plasticity of their expression, which is in agreement with the phenotypic data.

## Conclusions

The present work improves our understanding of the complex genetic architecture of bud set in poplar and of the considerable effect of G × E interaction in this process. QTL for bud set were identified and the projection of the most robust QTL on the *P. trichocarpa *genome, associated to previous gene expression studies, allowed the identification of CGs underlying the QTL. Furthermore, by investigating the physiological dynamics of bud set, we were able to confirm that the onset of growth cessation was triggered by night length, and the effect of environmental factors increased during the process. Additionally, a low number of plastic genotypes was responsible for the observed G × E interaction, with some of them being sensitive to temperature for the onset of growth cessation.

The recent development of the draft genome sequencing projects of *P. nigra *(Morgante & Zaina, personal communication), as well as the whole-genome SNP annotation, and the high throughput SNP genotyping platform (Faivre-Rampant & Zaina, personal communication) will be valuable tools allowing the scanning of allelic variation in CGs and analysis of natural populations by association mapping to determine the alleles associated with bud set traits. While some progress has been made in mapping bud set QTL, the elucidation of the underlying molecular mechanisms remains a bottleneck. Therefore, using expression QTL (eQTL) analysis will help to identify genes and gene networks associated to bud phenology and underlying traits involved in growth. This study opens up stimulating perspectives for molecular breeding aimed at increasing forest adaptation and productivity in a rapidly changing environment.

## Methods

### Plant material

A full-sib family of *P. nigra *(POP5) was used to score phenological characters and map QTL. The QTL mapping pedigree, already used in a previous genetic mapping study [29], consisted of 162 genotypes and derived from an intraspecific controlled cross between genotype '58-861' (female) from Val Cenischia (northern Italy; 45°09'N, 07°01'E) and genotype 'Poli' (male) from Policoro (southern Italy; 40°09'N, 16°41'E). The parents originate from contrasting environments and have highly divergent phenotypes [54].

### Experimental sites

The study was conducted at two sites in northern (CV, 44°42'N, 07°41'E) and central Italy (VT, 42°25'N, 12°05'E) at an elevation of 285 m and 310 m above sea level, respectively. A third experimental plantation including seven randomly collected genotypes of POP5 and the two parents, was established in 2008 in northern Italy (SAV, 44°36'N, 07°37'E). The soil is alluvial with sandy loam texture in CV and SAV, and sandy silt in VT. Annual precipitation and temperature averages of the years of study were 603 mm and 11.5°C (CV), 1125 mm and 14.2°C (VT), and 968 mm and 12.2°C (SAV). During the period of measurements (August, 1^st ^to October, 31^st^), rainfall in VT was lower than in CV and SAV (152.2 mm vs. 313.4 mm vs. 195.6 mm), while the average daily minimum temperature was higher in VT (13.4°C) as compared to CV and SAV (11.7°C) (Additional file 7: Figure S3). CNL, using sunrise and sunset times retrieved from the United States Naval Observatory http://aa.usno.navy.mil/, was calculated from the July, 1^st ^as a starting date. The same date was used for the calculation of CMT_10_. In this case, the temperature value below 10°C was subtracted to 10, giving to lower temperatures a larger contribution to the parameter.

### Experimental design

Three experimental plantations were established in April 2003 (CV) and April 2008 (VT and SAV) from hardwood cuttings (25 cm long) planted at a distance of 2 × 0.75 m. The SAV experiment was used to reinforce the observations of phenotypic plasticity of the parents and POP5 genotypes. The field trials were established using a completely randomized block design with six blocks in CV and SAV and five blocks in VT. One ramet per genotype and parent was randomly assigned to each block. A double border row using clonal material of the same species was planted around the experimental plantations to reduce border effects. The sites were regularly irrigated and treated with pesticides as necessary throughout the growing season. Trees were coppiced at the end of the second growing season (February 2005) in CV and the first growing season (February 2009) in VT and SAV. The re-sprouts were thinned to a single stem.

### Phenotypic data collection

#### Bud set

**Figure 6:**
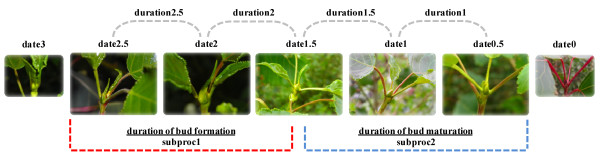
**High-resolution scoring system for bud set process in *Populus nigra***. date3: more than 2 rolled-up leaves; date2.5: last leaves at the same height, last leaves still rolled-up; date2: last leaves at the same height, last leaf (partially) rolled-up, other leaves fully stretched; date1.5: bud scales first visible, color of second last leaf comparable to older leaves, last leaf partially rolled; date1: bud well visible, bud scales predominantly green, all leaves are stretched; date0.5: bud scales green to red, stipules of last leaves subtending the bud are green; date0: apical bud red-brown. Duration of bud set stages (duration2.5 to duration1), duration of bud formation (subproc1) and bud maturation (subproc2) were indirectly estimated. Pictures were taken from individuals of the *P. nigra *mapping pedigree during phenological sampling. The scoring system for bud set process was adapted and modified from Rohde et al. [9].

Phenological measurements were taken in autumn 2005 in CV and data were collected every two days from September 9 (DOY 252) to October 13 (DOY 286). In VT, bud set was scored in autumn 2008 and data were collected every three days, from September 15 (DOY 259) to October 20 (DOY 294). In SAV bud set was scored every ten days in 2009, from August 18 (DOY 230) to October 7 (DOY 280). All trees showing abnormal apical behavior biasing the natural phenological process were excluded from further measurements. The timing of six of the seven discrete phenological stages (from the active growth (date3) to the end of bud maturation (date0.5)) were assessed by visual inspection as described in Rohde et al. [9] with a slight modification to the protocol as illustrated in Figure 6. Since date0 and date0.5 differ only in the color of the bud and were therefore difficult to be accurately measured, the former has been excluded in the current study. Data were smoothed using a local polynomial regression of degree 2 [55], with the different dates of observation as predictors, to estimate stages that had not been observed in the field because of the speed of the phenological process (*R^2 ^*ranging from 0.964 to 0.999). From the fitted curve, discrete values for DOY were retrieved for all stages from 3 to 0.5, respecting the range of observed stages. Complementary traits such as duration of different stages and the length of the subprocess to complete bud formation (subproc1) and bud maturation (subproc2) were indirectly estimated (Figure 6). This new scoring system allows the measurement of all phenotypic aspects of growth cessation and bud set. The dataset from CV was statistically re-analyzed (data modified from Rohde et al. [9]) to enlarge the number of genotypes to be used for G × E analyses and the evaluation of phenotypic plasticity in the five discrete phenological stages. Bud set was recorded on 148 genotypes at CV and 154 genotypes at VT, with at least three replicates. The different number of genotypes was attributed to mortality.

#### Growth

Annual growth, expressed as the total stem height (cm) and circumference (mm), was measured at the end of the growing season (January 2006 at CV and December 2008 at VT). Stem height was measured with an accuracy of one cm using an extendable aluminum pole. Stem circumference was measured at 1 m above ground level to the nearest mm using a tape. These measurements were used as covariates to assess their influence on the phenotypic variation of the bud set process.

### Statistical analyses

Data were analyzed using SAS software (SAS Institute, Cary, NC). Analyses of variance within and between sites, broad-sense heritability estimation at individual and genotypic level (H_ind_^2^, H_gen_^2^) and Pearson correlations among traits were calculated as described in Dillen et al. [56]. The estimation of variance components was obtained using the restricted maximum likelihood (REML) procedure. Genetic correlations between traits, for each site, were calculated using *r_g _*= *σ_G(xy)_*/[*σ^2^_G(x) _*× *σ^2^_G(y)_*]^1/2^, where *σ^2^_G(x) _*is the genetic variance component for trait *x*, *σ^2^_G(y) _*is the genetic variance component for trait *y*, and *σ_G(xy) _*is the genetic covariance between *x *and *y *[57]. The genetic covariance *σ_G(xy) _*was calculated according to the formula *σ_G(xy) _*= [(*σ^2^_G(x+y)_*-*σ^2^_G(x)_*-*σ^2^_G(y)_*)/2] [34]. The variance of genetic correlation was calculated using *σ^2^(r_g_) *= (1-*r_g_^2^*)*^2^*/*n_c _*[58], where *n_c _*is the number of genotypes used to calculate the covariance component, and standard deviation as *SD *= [*σ^2^*(*r_g_*)]^1/2^.

The G × E interaction, including site and year effects, was investigated in the current study. When the analysis of variance showed significant G × E interaction (*P *≤ 0.05), the change in ranking of genotypes was evaluated by the Spearman rank coefficient (ρ) on genotypic means. In order to dissect the G × E interaction and to understand genotype contribution to this source of phenotypic variance, the Wricke's relative ecovalence at genotypic level (*W_i_^r^*) was used as a measure of the phenotypic plasticity of each genotype between environments [59].

Multivariate analyses using PCA were conducted on individual tree basis at each site to select traits that account for most of the variance in the observed phenological process before QTL analysis.

### QTL analyses

QTL analyses were performed on selected traits (date2.5, date1.5, subproc1 and subproc2 expressed as CNL), which resulted after PCA as the most discriminative in terms of phenotypic variation. Analyses were carried out as described in Rae et al. [60] and in Rohde et al. [9]. The framework maps of the pedigree, described in Gaudet et al. [29], were improved by addition of 62 F_1 _progenies to obtain a total of 154 individuals for QTL analysis. Data were processed with MultiQTL software http://www.multiqtl.com/ and the multiple environment approach was used to increase the accuracy of the estimated QTL position and effect [56,60]. The QTL presented in this study had a chromosome-wise statistical significance level of 0.05 and a bootstrap analysis with 1,000 data permutations was carried out to estimate the 95% confidence interval of the QTL. The different effect of the QTL between the two sites was tested according to Rae et al. [60].

### QTL projections

QTL were projected on the reference genome sequence of *P. trichocarpa*, using anchored markers. For each QTL, the SSRs nearest to the interval extremities and with known position on *P. trichocarpa *genome were used to calculate the relationship between the genetic and the physical distance [61] to obtain the physical coordinates of the QTL interval on the *P. trichocarpa *genome sequence. When two or more QTL co-located on the same genomic region, a unique overlap QTL interval was considered and a deep annotation of its sequence was done in terms of genes and SNP markers. The gene report was based on the U.S. Department of Energy Joint Genome Institute (JGI) v2.2 gene annotation of the *P. trichocarpa *genome assembly v2. The SNP report was based on the high-throughput re-sequencing of four *P. nigra *genotypes (Poli, 71077-308, BEN3, and BDG), which provided a genome-wide SNP detection, displayed through the IGA gbrowse tool [30].

### Candidate gene selection

A bibliographical review was conducted to identify genes or gene models putatively associated with phenology [6,9,52] in *Populus*. Keywords such as phenology, bud set, flowering time pathway, circadian-clock genes and photoperiod-related genes were used for a web-based search in PubMed and GenBank of the National Center for Biotechnology Information (NCBI) http://www.ncbi.nlm.nih.gov/. A key source of CGs was represented by Simpson & Dean [62] and Ruttink et al. [31]. Then, the genes identified from the resulting literature collection were queried in the *P. trichocarpa *genome browser (JGI), assembly v2. When the query belonged to a different *genus *than *Populus*, a TBLASTX search was used; otherwise a BLASTN search was applied. The LG and base pair (bp) location of each gene model producing a statistically significant alignment with the query sequence was compared to the QTL intervals previously projected on the genome. Annotated genes known to be involved in bud set or development (or family members of such genes) that fell within these intervals were considered to be positional CGs. Their functionality was confirmed by searching the *P. trichocarpa *ESTs database http://www.populus.db.umu.se/index.html[63] in order to have an additional support in the discovery of the most promising CGs.

## Competing interests

The authors have no business relationships that could be construed as a potential conflict of interest.

## Authors' contributions

FF and MS participated in the design and performance of the experiments, statistical analyses, data interpretation and writing of the paper; NM co-performed phenological experiments; MG carried out QTL data analyses; MG, GZ and MM performed candidate gene identification, and participated in writing of the paper; AH and IB participated in data analyses and interpretation, and writing of the paper. MS, CB and GSM conceived and supervised the study. All authors jointly interpreted the results, read and approved the final manuscript.

## Supplementary Material

Additional file 1 **Figure S1**. (Portable Document Format file) Photoperiod progression in the two experimental sites in Italy: Cavallermaggiore (CV) and Viterbo (VT). (a) The yearly variation in photoperiod at the two sites situated at 44°N (CV, red full lines) and 42°N (VT, black dotted lines), as well as for 35°N and 50°N of latitude. The gray lines correspond to the period of measurements of bud set process. (b) Cumulative night length (CNL) was calculated from July 1st in CV (red full lines) and VT (black dotted lines). Click here for file

Additional file 2 **Figure S2**. (Portable Document Format file) Linear regression between growth and selected phenological traits. Data were obtained from a *Populus nigra *full-sib family (POP5) grown in two sites in Italy: Cavallermaggiore (CV) and Viterbo (VT). Click here for file

Additional file 3 **Table S1**. (Portable Document Format file) Genetic variation in a *Populus nigra *full-sib family (POP5) grown in Cavallermaggiore (CV) in Italy. Parental values (i.e. female parent '58-861' mean ± standard error (SE)), family values (i.e. population means ± SE and level of significance differences between F_1 _genotypes) and genetic parameters (i.e. coefficient of genetic (CV_g_) and residuals (CV_ε_) variation and broad-sense heritability at individual (H_ind_^2^) and genotypic (H_gen_^2^) level ± SE). The *F*-test between parents were not performed because of the absence of 'Poli' (see Materials and Methods). Click here for file

Additional file 4 **Table S2**. (Portable Document Format file) Genetic variation in a *Populus nigra *full-sib family (POP5) grown in Viterbo (VT) in Italy. Parental values (i.e. female parent '58-861' and male parent 'Poli' means ± standard error (SE) and level of significance difference between the two), family values (i.e. population means ± SE and level of significance differences between F_1 _genotypes) and genetic parameters (i.e. coefficient of genetic (CV_g_) and residuals (CV_ε_) variation and broad-sense heritability at individual (H_ind_^2^) and genotypic (H_gen_^2^) level ± SE). The significance level of the *F*-test between the two parents for each trait is indicated as: *ns*, non significant; *, *P *≤ 0.05; **, *P *≤ 0.01; ***, *P *≤ 0.001. Click here for file

Additional file 5 **Table S3**. (Microsoft Excel file) List of candidate genes (CGs) from bibliography sources. Click here for file

Additional file 6 **Table S4**. (Portable Document Format file) Gene models search relating to bud set. Gene models in *Populus trichocarpa *genome sequence closest to the logarithm of the odds (LOD) peak of selected quantitative trait loci (QTL) for bud set traits found in *P. nigra*. Gene models were searched in the sequence at ± 10 Kbp around the position of LOD peak or ± 30 Kbp to find at least one gene per LOD peak. LG, linkage group on which QTL were found. bp, base pairs. Click here for file

Additional file 7 **Figure S3**. (Portable Document Format file) Meteorological characteristics of the three experimental sites taken into account for phenotypic plasticity. Data were obtained from nearby meteorological stations in the three experimental sites in Italy, Cavallermaggiore (CV), Viterbo (VT) and Savigliano (SAV), where the *Populus nigra *full-sib family (POP5) was studied. Click here for file
