# Supplementary material for: Phenotypic plasticity, QTL mapping and genomic characterization of bud set in black poplar
Source: BMC Plant Biol. 2012 Apr 3;12:47. doi: 10.1186/1471-2229-12-47 (PMC3378457; doi:10.1186/1471-2229-12-47)
Supplement: Additional file 1 — Figure S1. (Portable Document Format file) Photoperiod progression in the two experimental sites in Italy: Cavallermaggiore (CV) and Viterbo (VT). (a) The yearly variation in photoperiod at the two sites situated at 44°N (CV, red full lines) and 42°N (VT, black dotted lines), as well as for 35°N and 50°N of latitude. The gray lines correspond to the period of measurements of bud set process. (b) Cumulative night length (CNL) was calculated from July 1st in CV (red full lines) and VT (black dotted lines). [file 1471-2229-12-47-S1.PDF]

Additional file 1: Photoperiod progression in the two experimental sites in Italy: Cavallermaggiore (CV) and Viterbo (VT).

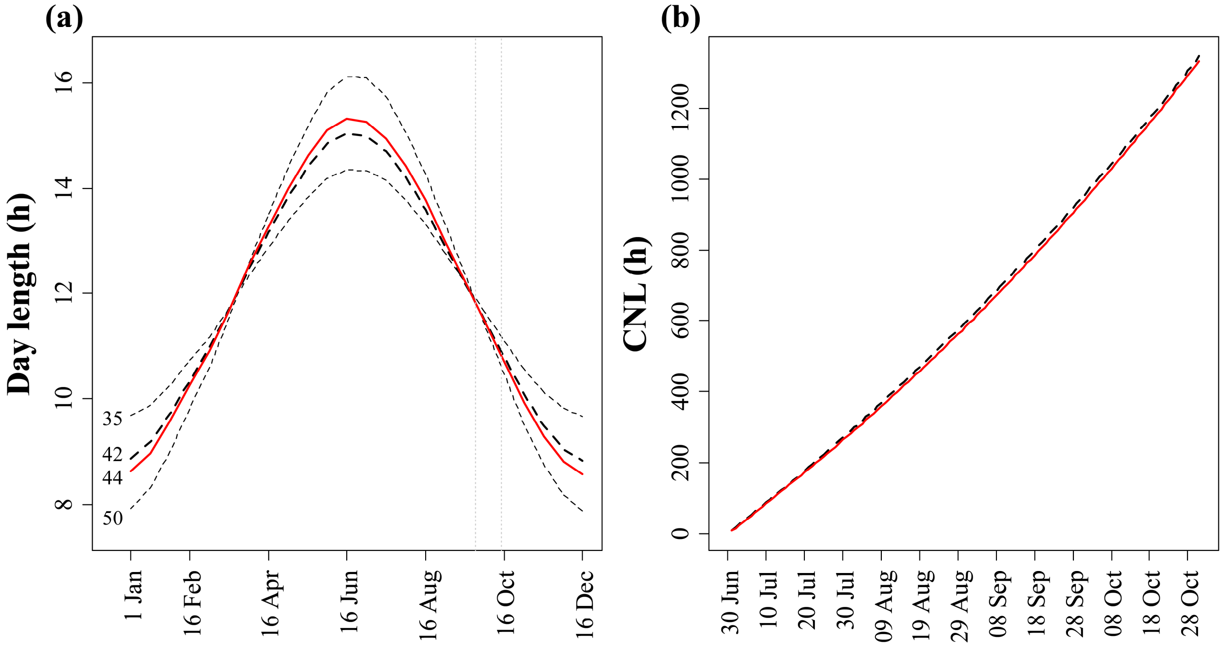

Figure S1 Photoperiod progression in the two experimental sites in Italy: Cavallermaggiore (CV) and Viterbo (VT). (a) The yearly variation in photoperiod at the two sites situated at 44°N (CV, red full lines) and 42°N (VT, black dotted lines), as well as for 35°N and 50°N of latitude. The gray lines correspond to the period of measurements of bud set process. (b) Cumulative night length (CNL) was calculated from July 1st in CV (red full lines) and VT (black dotted lines).
