# Supplementary material for: Phenotypic plasticity, QTL mapping and genomic characterization of bud set in black poplar
Source: BMC Plant Biol. 2012 Apr 3;12:47. doi: 10.1186/1471-2229-12-47 (PMC3378457; doi:10.1186/1471-2229-12-47)
Supplement: Additional file 2 — Figure S2. (Portable Document Format file) Linear regression between growth and selected phenological traits. Data were obtained from a Populus nigra full-sib family (POP5) grown in two sites in Italy: Cavallermaggiore (CV) and Viterbo (VT). [file 1471-2229-12-47-S2.PDF]

Additional file 2: Linear regression between growth and selected phenological traits.

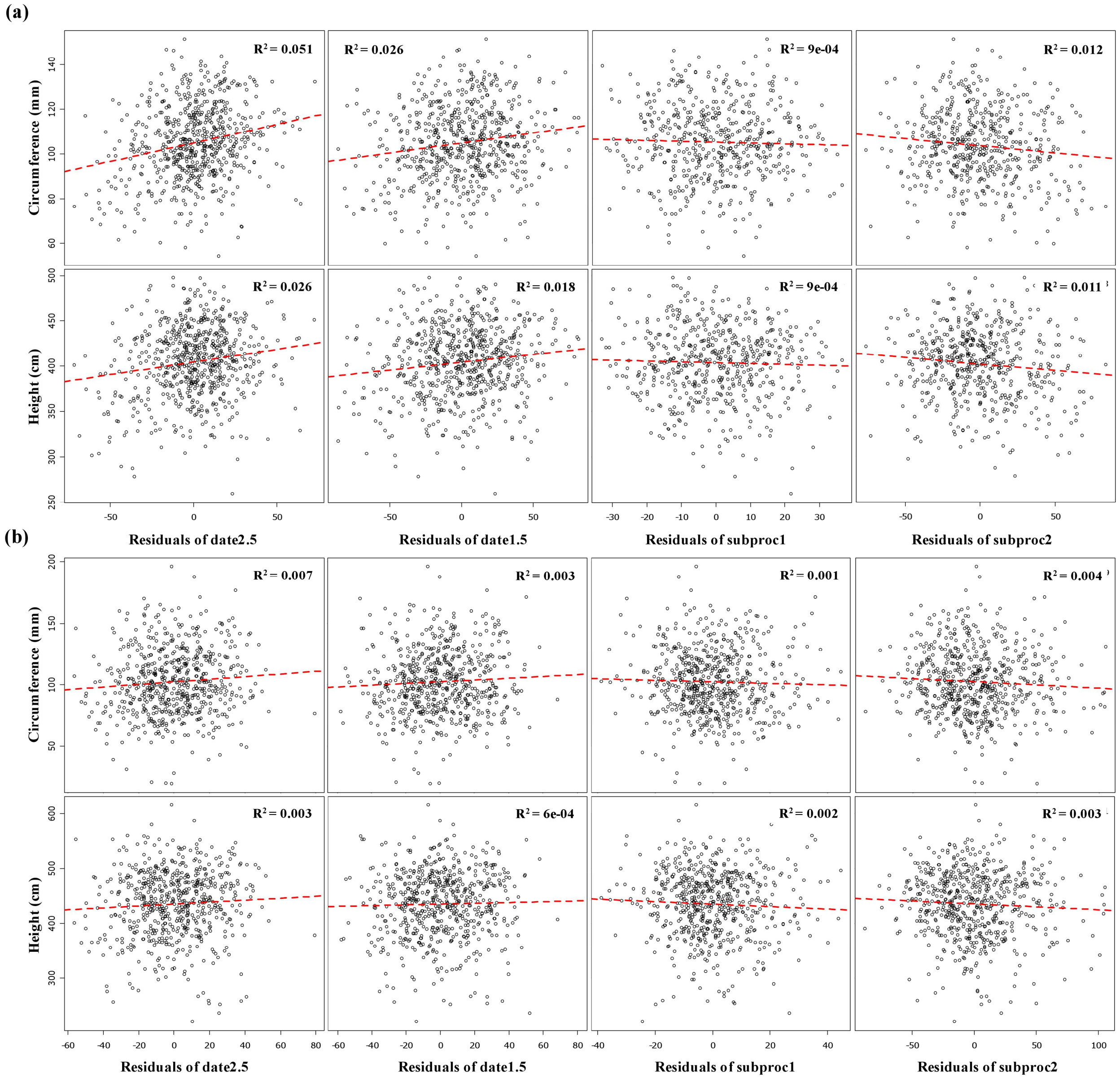

Figure S2 Linear regression between growth and selected phenological traits. Data were obtained from a *Populus nigra* full-sib family (POP5) grown in two sites in Italy: Cavallermaggiore (CV) and Viterbo (VT).
