# Supplementary material for: Phenotypic plasticity, QTL mapping and genomic characterization of bud set in black poplar
Source: BMC Plant Biol. 2012 Apr 3;12:47. doi: 10.1186/1471-2229-12-47 (PMC3378457; doi:10.1186/1471-2229-12-47)
Supplement: Additional file 3 — Table S1. (Portable Document Format file) Genetic variation in a Populus nigra full-sib family (POP5) grown in Cavallermaggiore (CV) in Italy. Parental values (i.e. female parent '58-861' mean ± standard error (SE)), family values (i.e. population means ± SE and level of significance differences between F1 genotypes) and genetic parameters (i.e. coefficient of genetic (CVg) and residuals (CVε) variation and broad-sense heritability at individual (Hind2) and genotypic (Hgen2) level ± SE). The F-test between parents were not performed because of the absence of 'Poli' (see Materials and Methods). [file 1471-2229-12-47-S3.PDF]

Additional file 3: Genetic variation in a *Populus nigra* full-sib family (POP5) grown in Cavallermaggiore (CV) in Italy.

Table S1 Genetic variation in a *Populus nigra* full-sib family (POP5) grown in Cavallermaggiore (CV) in Italy. Parental values (i.e. female parent ‘58-861’ mean ± standard error (SE)), family values (i.e. population means ± SE and level of significance differences between F_1 genotypes) and genetic parameters (i.e. coefficient of genetic (CV_g) and residuals ($CV_ε$) variation and broad-sense heritability at individual (H^2_{ind}) and genotypic (H^2_{gen}) level ± SE). The *F*-test between parents were not performed because of the absence of ‘Poli’ (see Materials and Methods).

CV		Parental values						Family values			Genetic values						
Trait	Climatic parameter	Poli	± SE	58-861	±	SE	*F* test	General mean	±	SE	P	CV_g (%)	$CV_ε$ (%)	H^2_{ind}	± SE	H^2_{gen}	± SE
date2.5	CNL	-	-	781.17	±	10.16	-	864.23	±	1.62	***	3.46	3.24	0.53	± 0.04	0.84	± 0.02
date2	CNL	-	-	824.17	±	9.47	-	913.87	±	1.68	***	3.25	3.35	0.48	± 0.05	0.81	± 0.02
date1.5	CNL	-	-	863.93	±	9.51	-	964.08	±	1.76	***	2.96	3.56	0.40	± 0.05	0.75	± 0.03
date1	CNL	-	-	934.33	±	20.75	-	1019.04	±	1.64	***	2.45	3.29	0.35	± 0.05	0.70	± 0.03
date0.5	CNL	-	-	1087.67	±	9.46	-	1098.25	±	0.63	***	0.53	1.37	0.14	± 0.04	0.43	± 0.05
duration2.5	CNL	-	-	43.00	±	2.49	-	49.64	±	0.58	***	7.78	26.76	0.11	± 0.04	0.36	± 0.05
duration2	CNL	-	-	39.76	±	3.56	-	50.21	±	0.60	***	12.99	28.09	0.18	± 0.04	0.49	± 0.04
duration1.5	CNL	-	-	70.40	±	14.61	-	54.95	±	0.58	***	11.59	24.58	0.23	± 0.04	0.57	± 0.04
duration1	CNL	-	-	153.34	±	17.27	-	79.95	±	1.43	***	23.32	38.61	0.27	± 0.04	0.62	± 0.04
subproc1	CNL	-	-	82.76	±	5.43	-	99.85	±	1.02	***	10.01	23.59	0.15	± 0.04	0.44	± 0.05
subproc2	CNL	-	-	223.74	±	2.95	-	134.91	±	1.59	***	16.70	24.59	0.32	± 0.05	0.67	± 0.04
