# Supplementary material for: Phenotypic plasticity, QTL mapping and genomic characterization of bud set in black poplar
Source: BMC Plant Biol. 2012 Apr 3;12:47. doi: 10.1186/1471-2229-12-47 (PMC3378457; doi:10.1186/1471-2229-12-47)
Supplement: Additional file 4 — Table S2. (Portable Document Format file) Genetic variation in a Populus nigra full-sib family (POP5) grown in Viterbo (VT) in Italy. Parental values (i.e. female parent '58-861' and male parent 'Poli' means ± standard error (SE) and level of significance difference between the two), family values (i.e. population means ± SE and level of significance differences between F1 genotypes) and genetic parameters (i.e. coefficient of genetic (CVg) and residuals (CVε) variation and broad-sense heritability at individual (Hind2) and genotypic (Hgen2) level ± SE). The significance level of the F-test between the two parents for each trait is indicated as: ns, non significant; *, P ≤ 0.05; **, P ≤ 0.01; ***, P ≤ 0.001. [file 1471-2229-12-47-S4.PDF]

Additional file 4: Genetic variation in a *Populus nigra* full-sib family (POP5) grown in Viterbo (VT) in Italy.

Table S2 Genetic variation in a *Populus nigra* full-sib family (POP5) grown in Viterbo (VT) in Italy. Parental values (i.e. female parent '58-861' and male parent 'Poli' means ± standard error (SE) and level of significance difference between the two), family values (i.e. population means ± SE and level of significance differences between F_1 genotypes) and genetic parameters (i.e. coefficient of genetic (CV_g) and residuals (CV_ε) variation and broad-sense heritability at individual (H^2_{ind}) and genotypic (H^2_{gen}) level ± SE). The significance level of the *F*-test between the two parents for each trait is indicated as: *ns*, non significant; *, $P \leq 0.05$; **, $P \leq 0.01$; ***, $P \leq 0.001$.

VT		Parental values							Family values				Genetic values							
Trait	Climatic parameter	Poli	±	SE	58-861	±	SE	*F* test	General mean	±	SE	P	CV_g (%)	CV_ε (%)	H^2_{ind}	±	SE	H^2_{gen}	±	SE
date2.5	CNL	960.04	±	12.18	801.54	±	1.74	***	861.95	±	1.22	***	2.22	2.76	0.39	±	0.05	0.73	±	0.03
date2	CNL	1016.16	±	12.17	846.86	±	4.68	***	907.51	±	1.22	***	2.17	2.59	0.40	±	0.05	0.74	±	0.03
date1.5	CNL	1076.22	±	9.47	906.56	±	8.68	***	950.24	±	1.28	***	2.16	2.62	0.39	±	0.05	0.73	±	0.03
date1	CNL	1138.32	±	8.85	968.12	±	8.06	***	1001.44	±	1.45	***	2.02	3.04	0.30	±	0.04	0.64	±	0.04
date0.5	CNL	1202.04	±	7.63	1053.68	±	3.24	***	1093.54	±	2.22	***	1.71	3.96	0.16	±	0.04	0.45	±	0.04
duration2.5	CNL	56.12	±	3.03	45.32	±	3.12	*	45.56	±	0.32	**	6.16	19.90	0.09	±	0.04	0.28	±	0.04
duration2	CNL	59.76	±	4.16	59.70	±	6.33	*Ns*	42.73	±	0.38	*	5.58	21.46	0.07	±	0.03	0.24	±	0.04
duration1.5	CNL	62.40	±	8.83	61.56	±	3.04	*Ns*	51.20	±	0.58	*ns*	4.35	28.80	0.04	±	0.03	0.15	±	0.04
duration1	CNL	63.72	±	2.24	85.56	±	11.11	*Ns*	92.10	±	2.11	*ns*	3.68	28.59	0.03	±	0.03	0.10	±	0.04
subproc1	CNL	116.18	±	4.66	105.02	±	8.14	*Ns*	88.29	±	0.66	**	5.03	17.43	0.08	±	0.04	0.27	±	0.04
subproc2	CNL	126.12	±	9.79	147.12	±	11.91	*Ns*	143.31	±	2.17	*ns*	4.19	25.37	0.04	±	0.03	0.15	±	0.04
