# Supplementary material for: Phenotypic plasticity, QTL mapping and genomic characterization of bud set in black poplar
Source: BMC Plant Biol. 2012 Apr 3;12:47. doi: 10.1186/1471-2229-12-47 (PMC3378457; doi:10.1186/1471-2229-12-47)
Supplement: Additional file 6 — Table S4. (Portable Document Format file) Gene models search relating to bud set. Gene models in Populus trichocarpa genome sequence closest to the logarithm of the odds (LOD) peak of selected quantitative trait loci (QTL) for bud set traits found in P. nigra. Gene models were searched in the sequence at ± 10 Kbp around the position of LOD peak or ± 30 Kbp to find at least one gene per LOD peak. LG, linkage group on which QTL were found. bp, base pairs. [file 1471-2229-12-47-S6.PDF]

Additional file 6: Gene models search relating to bud set.

Table S4 Gene models search relating to bud set. Gene models in *Populus trichocarpa* genome sequence closest to the logarithm of the odds (LOD) peak of selected quantitative trait loci (QTL) for bud set traits found in *P. nigra*. Gene models were searched in the sequence at ±10 Kbp around the position of LOD peak or ±30 Kbp to find at least one gene per LOD peak. LG, linkage group on which QTL were found. bp, base pairs.

Trait_LG	LOD peak	v2.2 gene predicted ID	Chromosome	Start (bp)	Description	Molecular function	EST data	Phenology-related tissue/stage	Arabidopsis homolog
date2.5_I	5250740	POPTR_0001s06870	scaffold_1	5245963	RBR1 transcription factor binding	transcription factor binding	Yes	*Populus* flower; mixture of leaf, bud, stem, root *Populus*	AT3G12280
date1.5_I	5709370	POPTR_0001s07570	scaffold_1	5736117	unknown protein	unknown	Yes	mixture of leaf, bud, stem, root *Populus*	AT1G79660
		POPTR_0001s07580	scaffold_1	5738137	CCR4-NOT transcription complex protein	3'-5' exonuclease and ribonuclease activity	No	Not Applicable	AT5G22250
subproc2_I	6183036	POPTR_0001s08130	scaffold_1	6189291	ACR8; amino acid binding	amino acid binding	Yes	*Ricinus communis* flowers	AT1G12420
date2.5_IV	14458250	POPTR_0004s13990	scaffold_4	14460446	50S RIBOSOMAL PROTEIN L14-RELATED	structural constituent of ribosome	Yes	Any Related	ATCG00780
		POPTR_0004s14020	scaffold_4	14479313	CUL4 (CULLIN4) protein binding ubiquitin-protein ligase	protein binding	Yes	*Populus* apical shoot; mixture of leaf, bud, stem, root *Populus*	AT5G46210
date1.5_IV	7211363	POPTR_0004s08650	scaffold_4	7210228	ATMYB6; DNA binding / transcription factor	DNA binding	Yes	*Populus* apical shoot; *Populus* flower; mixture of leaf, bud, stem, root *Populus*	AT4G09460
date2.5_X<br>date1.5_X	4895589	POPTR_0010s04033	scaffold_10	4870271	nucleic acid binding; zinc ion binding	nucleic acid binding, zinc ion binding	No	Not Applicable	AT2G01050
date2.5_XIII	10113398	POPTR_0013s10120	scaffold_13	10113683	AMK2 ATP binding	ATP binding	Yes	*Populus* flower; *Populus* apical shoot;	AT5G47840

								mixture of leaf, bud, stem, root *Populus*	
		POPTR_0013s10130	scaffold_13	10118399	CCR4 kinase	kinase activity,	Yes	Any Related	AT5G47850
date2.5_XVI	3078766	POPTR_0016s05070	scaffold_16	3070224	UVI4 (UV-B-INSENSITIVE 4)	protein binding	Yes	*Populus* flower ; mixture of leaf, bud, stem, root *Populus*	AT2G42260
		POPTR_0016s05080	scaffold_16	3076201	RABA4D GTP binding	GTP binding	Yes	mixture of leaf, bud, stem, root *Populus*	AT3G12160
		POPTR_0016s05100	scaffold_16	3086565	basic helix-loop-helix (bHLH) family protein	DNA binding	Yes	flower, leaf and root non-normalized *Vitis vinifera*	AT2G42280
date1.5_XVI	2822081	POPTR_0016s04720	scaffold_16	2813838	DC1 domain-containing protein	zinc ion binding	Yes	mixture of leaf, bud, stem, root *Populus*	AT5G22355
		POPTR_0016s04730	scaffold_16	2819836	DC1 domain-containing protein		Yes	mixture of leaf, bud, stem, root *Populus*	AT2G44380
		POPTR_0016s04740	scaffold_16	2828231	DC1 domain-containing protein	zinc ion binding	Yes	mixture of leaf, bud, stem, root *Populus*	AT5G22355
date2.5_XVII	12215613	POPTR_0017s12070	scaffold_17	12212347	structural constituent of ribosome	structural constituent of ribosome	Yes	*Ricinus communis* flowers; mixture of leaf, bud, stem, root *Populus*	AT3G01170
date1.5_XVII	11574009	POPTR_0017s11760	scaffold_17	11585740	transcription regulator; BNQ1,	DNA binding	Yes	Any Related	AT5G39860
