# Supplementary material for: Phenotypic plasticity, QTL mapping and genomic characterization of bud set in black poplar
Source: BMC Plant Biol. 2012 Apr 3;12:47. doi: 10.1186/1471-2229-12-47 (PMC3378457; doi:10.1186/1471-2229-12-47)
Supplement: Additional file 7 — Figure S3. (Portable Document Format file) Meteorological characteristics of the three experimental sites taken into account for phenotypic plasticity. Data were obtained from nearby meteorological stations in the three experimental sites in Italy, Cavallermaggiore (CV), Viterbo (VT) and Savigliano (SAV), where the Populus nigra full-sib family (POP5) was studied. [file 1471-2229-12-47-S7.PDF]

**Additional file 7: Meteorological characteristics of the three experimental sites taken into account for phenotypic plasticity.**

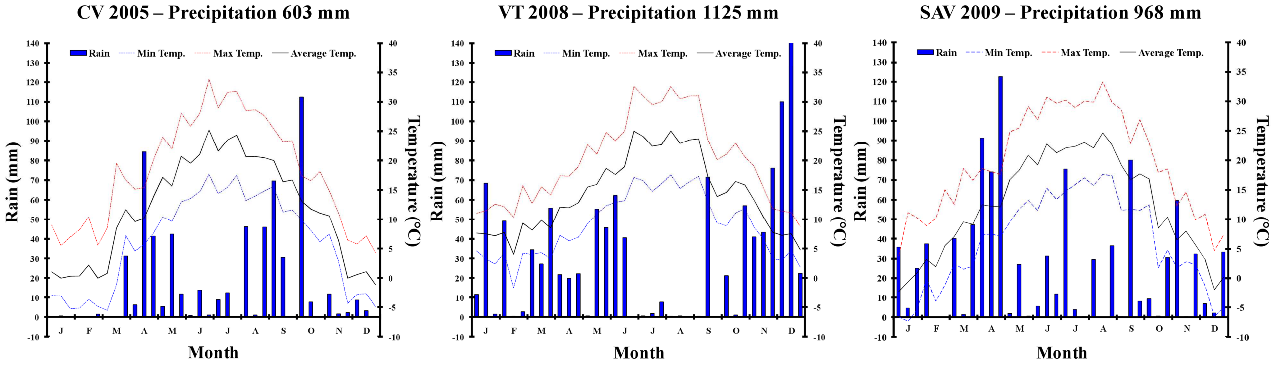


**Figure S3 Meteorological characteristics of the three experimental sites taken into account for phenotypic plasticity.** Data were obtained from nearby meteorological stations in the three experimental sites in Italy, Cavallermaggiore (CV), Viterbo (VT) and Savigliano (SAV), where the *Populus nigra* full-sib family (POP5) was studied.
